# Multi-Task Classification for Improved Health Outcome Prediction Based on Environmental Indicators

**DOI:** 10.1109/access.2023.3295777

**Published:** 2023-07-14

**Authors:** MITRA ALIREZAEI, QUYNH C. NGUYEN, ROSS WHITAKER, TOLGA TASDIZEN

**Affiliations:** 1 Department of Electrical and Computer Engineering, University of Utah, Salt Lake City, UT 84112, USA; 2 Department of Epidemiology and Biostatistics, University of Maryland School of Public Health, College Park, MD 20742, USA; 3 School of Computing, University of Utah, Salt Lake City, UT 84112, USA

**Keywords:** Built environment, deep neural networks, Google street view, health outcomes, multi-task model

## Abstract

This paper aims to address the challenges associated with evaluating the impact of neighborhood environments on health outcomes. Google street view (GSV) images provide a valuable tool for assessing neighborhood environments on a large scale. By annotating the GSV images with labels indicating the presence or absence of specific neighborhood features, we can develop classifiers capable of automatically analyzing and evaluating the environment. However, the process of labeling GSV images to analyze and evaluate the environment is a time-consuming and labor-intensive task. To overcome these challenges, we propose using a multi-task classifier to enhance the training of classifiers with limited supervised GSV data. Our multi-task classifier utilizes readily available, inexpensive online images collected from Flickr as a related classification task. The hypothesis is that a classifier trained on multiple related tasks is less likely to overfit to small amounts of training data and generalizes better to unseen data. We leverage the power of multiple related tasks to improve the classifier’s overall performance and generalization capability. Here we show that, with the proposed learning paradigm, predicted labels for GSV test images are more accurate. Across different environment indicators, the accuracy, *F*_1_ score and balanced accuracy increase up to 6 % in the multi-task learning framework compared to its single-task learning counterpart. The enhanced accuracy of the predicted labels obtained through the multi-task classifier contributes to a more reliable and precise regression analysis determining the correlation between predicted built environment indicators and health outcomes. The *R*^2^ values calculated for different health outcomes improve by up to 4 % using multi-task learning detected indicators.

## I. INTRODUCTION

The *built environment* refers to the physical structure and features of a habitable area, including sidewalks, buildings, crosswalks, chain-link fences, parks, green spaces, and streetlights, that constitute the immediate, local environment for an area’s residents. The built environment plays an important role in shaping the health-related behaviors and exposures of its residents and, as a result, their health outcomes [1], [2].

The *physical disorder* of a built environment refers to the presence of signs and indicators that correspond to neglect, decay, and disruption. Such indicators include dilapidated buildings, garbage, graffiti, broken windows, chain-link fences, and abandoned houses. Physical disorder can have a negative impact on the health and overall well-being of residents. Research has shown that the physical disorder of a neighborhood can affect mental health, stress, depression, and rates of chronic disease [3], [4], [5].

Neighborhood features can affect health in various ways. A well-designed built environment can promote physical activity and healthy diets among residents. The presence of green spaces, parks, sidewalks, and access to healthy food can encourage exercise and recreational activities and result in a lower risk of obesity and diabetes [3], [6], [7]. Thus, examining the relationship between the built environment and health outcomes can help policymakers and public health professionals make informed decisions on how to enhance the built environment to improve health and well-being

Google Street View (GSV) images offer a valuable alternative to traditional in-person assessments of the built environment. Conventional methods require substantial resources, making it challenging to perform large-scale evaluations on a national level. However, with the use of GSV images, the need for expensive and time-consuming in-person surveys is reduced.

Although the process of accessing the built environment using GSV images still requires some level of effort and cost, deep learning networks have been utilized to automate this process in a more efficient and cost-effective way. The combination of GSV images and deep learning networks has the potential to transform the way in which the built environment is evaluated and analyzed. The use of deep learning networks enables the identification of important indicators in the images, the evaluation of neighborhoods based on those indicators, and the analysis of which indicators are correlated with health outcomes [3], [8], [9], [10].

Deep learning models consist of multiple neural layers and have demonstrated remarkable ability in handling complex tasks, including large-scale image classification (such as the 1000-class ImageNet dataset) [11], object detection [12], [13], image generation [14], [15], and natural language processing with models such as GPT [16], [17].

Despite their impressive performance, deep learning models typical require massive amounts of data for training because of the large number of parameters involved. For instance, the ImageNet dataset used for large-scale image classification contains over 14 million labeled images. The challenge of labeling large amounts of data remains a major obstacle to applying these models to new applications, such as detecting built environment indicators in GSV images. The process of manual labeling is costly, time-consuming, and not scalable for large datasets. In the absence of sufficiently large training datasets, neural networks with large numbers of free parameters tend to overfit the training data, leading to poor performance on previously unseen data.

Different approaches can be used to address limited data in deep learning models, depending on the specific task. In our project, we obtained images of indicators from Flickr database (which is free to access and download), avoiding the need for manual labeling. These images were then utilized to boost the accuracy of our primary objective, which is to classify GSV images in a multi-task learning framework [18], [19]. Our study demonstrates that the proposed approach results in a better accuracy of detecting environment indicators and a more precise estimation of correlated health outcomes.

## II. RELATED WORKS

### A. GOOGLE STREET VIEW IMAGES AND HEALTH OUTCOMES

Previously, neighborhood health indicators were evaluated through in-person neighborhood audits or surveys, which gather information about neighborhood characteristics [20], [21], [22], [23]. In-person neighborhood audits involve sending trained individuals to physically visit neighborhoods and collect data on various aspects such as cleanliness, safety measures, and social factors. Surveys conducted in the neighborhood involve contacting residents and gathering their perspectives through questionnaires or interviews. While these approaches can provide detailed information, they have limitations in terms of scalability, cost, and coverage, being able to assess only a small portion of neighborhoods.

Later, the use of GSV images was promoted as a way to assess built environment indicators on a large scale [3], [8], [9], [10], [24], [25], [26], [27]. GSV images offer a wealth of visual information about the built environment, including buildings, streets, sidewalks, green spaces, transportation infrastructure, and more on a large scale. The use of GSV images eliminates the need to physically visit different neighborhoods and can be done virtually.

In [24], the authors compared neighborhood measurements obtained through GSV images in 2008 and neighborhood audit data collected in 2007, and showed that GSV images can be used as an effective tool for auditing neighborhood environments. In [28], the authors developed a computer-assisted neighborhood visual assessment system (CANVAS) that uses GSV to conduct virtual audits of neighborhood environments. However, this system requires manual annotations of GSV images.

Adams et al. [29] utilized GSV images and developed deep learning models to detect small-scale features in urban environments that are related to pedestrian activity. Subsequently, Javanmardi et al. [3] performed virtual neighborhood audits using GSV images in regression analysis for chronic disease prevalence. The authors used available census tract data directly as targets for groups of images. Nguyen et al. [9] used GSV images and computer vision techniques with a logistic regression model to analyze cross-sectional associations with chronic health outcomes. Yue et al. [8] employed GSV images and a CNN network to investigate the relationship between built environments and chronic diseases and health behaviors. In another study, Nguyen et al. [10] performed a neighborhood built environment analysis for the state of Utah using GSV images.

The above mentioned computer vision models rely on a set of labeled data for training to detect built environment indicators. If the model is trained on insufficient data, it may not accurately detect built environment indicators, and the correlations estimated for health outcomes will not be reliable. Thus, developing a model that can be trained effectively with limited data is crucial in the identification of more complicated or subtle effects.

In this study, we aim to improve the classification model via multi-task learning and thus enhance the prediction of health outcomes correlated with predicted environment indicators on a national level.

### B. MULTI-TASK NETWORKS

To enhance the automation of accessing neighborhood characteristics and finding correlations with health, we utilize a multi-task learning framework.

Multi-task learning is the process of training a model on multiple tasks simultaneously and is way of improving generalization [18], [19], [30]. Two common ways of multi-task learning are learning via hard parameter sharing or via soft parameter sharing [18]. In hard parameter sharing [31], the model can be divided into two parts: 1) the task-specific part that is trained solely on examples specific to that task, and 2) the generic part that is shared across all different tasks [19]. Baxter et al. [32] showed that shared parameters improve generalization and reduce the generalization error bounds. The shared part of the model in multi-task learning leverages information and patterns learned from different tasks and improves the overall performance. Learning multiple tasks allows the model to generalize better and avoid overfitting to a single task, leading to better results overall [19]. Multi-task learning via soft parameter sharing is done by training separate models for each task. The distance between the models’ parameters is then regularized to keep the parameters similar [18].

Multi-task learning has been successfully applied in a variety of fields, such as natural language processing [33], speech recognition [34], and computer vision [12], [13]. In popular architectures such as FasterRCNN and YOLO [12], [13], the networks are trained using multiple objectives to predict both the class and the coordinates of an object within an image simultaneously.

Because the performance of multi-task networks depends on the weighting of the objectives, Kendall et al. [35] proposed a novel approach for determining the weighting of multiple losses in multi-task learning instead of manual tuning of the weights. Sener and Koltun [36] argued that multi-task learning, where tasks are solved by minimizing a linear combination of their individual losses, is effective only if the tasks do not compete with each other. Therefore, the authors cast multi-task learning as multi-objective optimization and show that their method outperforms models with multi-task learning formulations.

Multi-task learning can also be used when the focus is on performance for a single task. In such a scenario, selecting a related task as an auxiliary task within the multi-task learning framework can provide the benefits of multi-task learning [18]. As an example, Zhang et al. [37] improved detection through multi-task learning. They improved facial landmark detection by jointly optimizing for related tasks such as head pose estimation and facial attribute inference.

In this paper, we also have utilized multi-task learning to enhance the generalization for classifying GSV images. The classification network is trained on two tasks concurrently, where the second task serves to improve the optimization for the GSV task, leading to a more effective GSV classification network. In the following section, we will delve into the details of our proposed approach to improve detection of environment indicators and examine their impact on health outcomes.

## III. METHODS

In this section, we present an investigation of the classification of GSV images. Our objective is to train a classifier that can accurately detect built environment indicators affecting health outcomes.

The built environment indicators that we focus on in this study are:

1. the presence of dilapidated buildings, which is an indicator of a neighborhood with poverty or lack of maintenance and can have negative effects on the health and well-being of the community,
2. the presence of streetlights, which is a potential indicator of walkability and urban development,
3. the presence of chain-link fences, which usually correspond to neighborhoods with abandoned lots and buildings and is an indicator of neglect and physical disorder.

Nonetheless, some neighborhoods use chain-link fences around homes and their connection with health outcomes is less known. Emerging research suggests chain-link fences in residential areas lower property values, and some cities are prohibiting their use in residential areas. More research is needed to investigate associations between chain-link fences and health.

**FIGURE 1.**
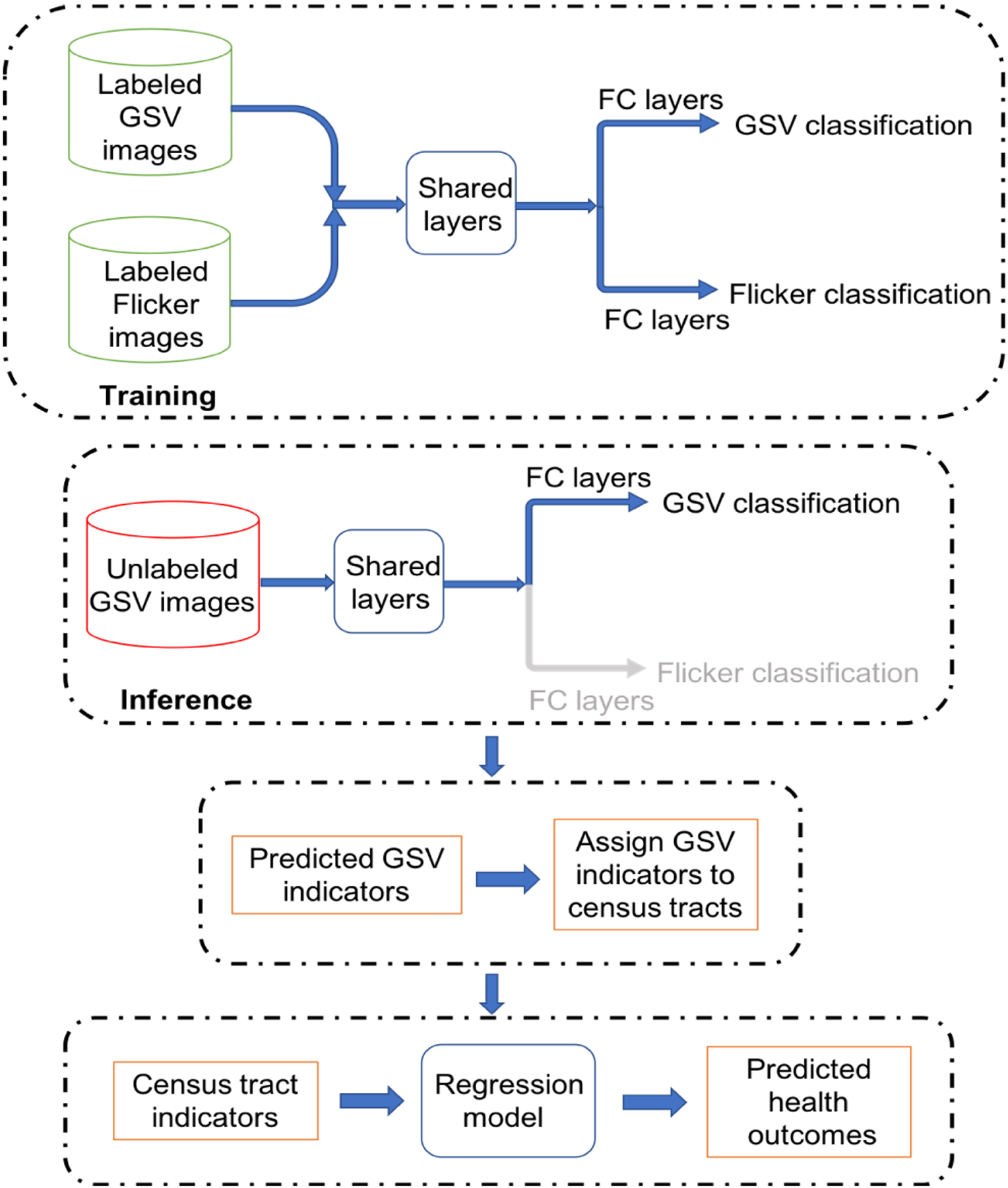
A multi-task classification model is trained on Flickr and GSV images, and then used to label test GSV images. The results are mapped to census-tract level, and a regression model is trained to predict health outcomes based on predicted indicators.

Fig. 1 shows the general overview of the proposed algorithm. First, a classification model is trained on Flickr and GSV images in a multi-task framework. Then, the trained classifier is used to label the test GSV images. The GSV images along their with predicted indicators are mapped to census-tract level. Finally, a regression model is trained to predict health outcomes using predicted indicators on the census-tract level.

In the following sections, we will explain the process of classifying GSV images using a single-task network, as well as the details of using multi-task classification to improve the classification results. We will also discuss using a regression model to examine the correlation between predicted environment indicators and health outcomes.

### A. THE SINGLE-TASK CLASSIFIER

In the context of this application, the single-task classifier is used to detect the presence or absence of each built environment indicator in an image. This task is a binary classification, and we have trained separate classifiers for detecting each built environment indicator. Each classifier is composed of two main components: a feature extractor and a classification layer.

The feature extractor consists of multiple convolutional layers that apply a set of filters to the input image, transforming it into a collection of feature maps that includes important patterns and structures. The extracted features are passed on to a classification layer. The classification layer consists of fully connected layers. The purpose of the fully connected layers is to map the extracted features to specific class labels. The output of the fully connected layers represents the likelihood of the presence or absence of a particular built environment indicator.

In this paper, we use a pretrained image feature extractor, the ResNet-18 network, which was trained on the ImageNet dataset. ResNet-18 consists of several convolutional layers and residual blocks. The residual blocks include residual connections that address the problem of vanishing gradients and facilitate the training of deeper networks.

Training a complex network like ResNet-18 from scratch on a limited set of labeled GSV images can be challenging. However, by using a pretrained network that has already learned useful features and representations from a large dataset such as ImageNet, we are able to leverage that knowledge as a starting point for the GSV classification task which leads to improved performance and faster convergence compared to training the network from scratch on the limited GSV dataset.

### B. THE MULTI-TASK CLASSIFIER

**FIGURE 2.**
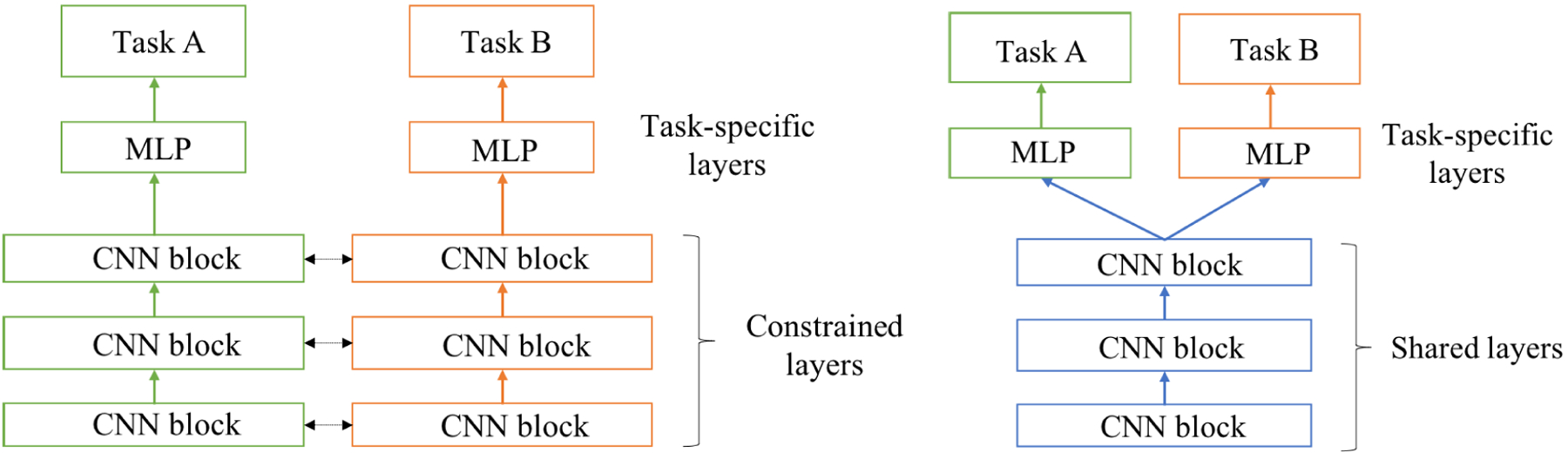
Multi-task learning using (Left) soft parameter sharing and (Right) hard parameter sharing. The model typically consists of CNN blocks followed by a multi-layer perceptron (MLP). The shared layers across different tasks are depicted in blue, while task-specific layers are shown in black and green for both methods [18].

In multi-task learning (MTL), a model is trained on different tasks simultaneously. Due to sharing representations between related tasks, the model learns general representations of data that are useful across different tasks, which results in improving generalization and reducing overfitting [19]. To further clarify the concept of multi-task learning, we include Fig. 2 that illustrates the comparison between soft parameter sharing and hard parameter sharing methods.

In the soft parameter sharing approach, each task has its own dedicated set of parameters, and the networks for both tasks are trained independently.

In contrast, in our chosen hard parameter sharing approach, we use a shared pretrained classifier, such as ResNet-18, as the backbone of the network. The parameters of this backbone are shared across both tasks. On top of the shared network, we have task-specific classification layers responsible for each task’s final predictions. This architecture allows the network to benefit from the shared representations learned by the pretrained classifier, leading to improved generalization and performance.

**FIGURE 3.**
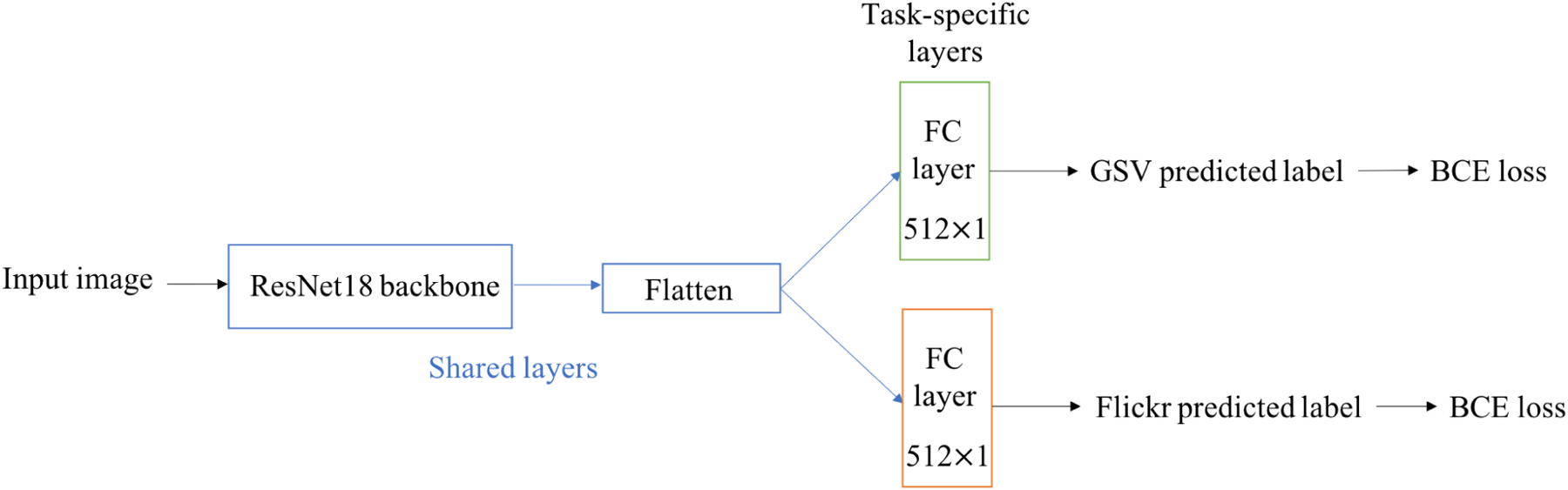
Our multi-task classifier using hard parameter sharing method.

Fig. 3 provides a visual representation of how multi-task classification is done using hard parameter sharing method.

Our multi-task classifier is trained to perform two tasks: classifying GSV images and classifying images collected from Flickr.^1^ Both sources have the same class labels. More information on how the dataset from Flickr is collected can be found in Section IV.

For each environment indicator, we gathered two sets of images, one set containing images where the indicator is present and another set containing images where the indicator is not present. Using the MTL approach in this case, the Flickr images are used to help the network learn features that are shared across GSV and Flickr images, helping the network learn robust and more crucial features leading to a better performance in classifying GSV images.

A pretrained classifier such as ResNet-18 is used as the shared part of the MTL network. Two additional classification layers are then added to the shared network, each one responsible for classifying a distinct task. The use of a multi-task network is expected to result in superior performance compared to a single-task network, as confirmed by experimental results in Section V.

After developing an effective classification model, the model is applied to label a large dataset of GSV images collected from various locations across the country. The predicted labels for each indicator are then utilized to estimate the relationship between environment indicators and health outcomes using a regression model, which will be discussed in the following section.

### C. REGRESSION MODELS FOR PREDICTING HEALTH OUTCOMES

In order to predict health outcomes associated with environment indicators, we have used fully connected neural networks that take as inputs environment indicators at the census-tract level and map them to associated health outcomes at the same tract level. The environment indicators are concatenated together and used as inputs for the model. The training inputs for the neural network regression models are obtained using the predictions of a previously trained classifier on unlabeled GSV test images consisting of 164 million images.

The GSV images, along with their predicted labels, are then mapped to census tracts based on their latitude and longitude information. For each census tract, the total number of images containing a given indicator is calculated, and this count is normalized by dividing it by the total number of images in that census tract. These normalized counts of environment indicators, calculated from the GSV images, are used as inputs for the regression model. The training outputs for the regression models are health outcomes at the census tract level, obtained from data sources such as the behavioral risk factor surveillance system (BRFSS).

The neural network regression models are optimized to minimize the difference between the predicted health outcomes and the ground truth health outcomes. The trained models are then be used to predict health outcomes for new census tracts based on the environment indicators present in those areas.

## IV. DATA COLLECTION

### A. GSV DATA COLLECTION AND LABELING

We used 164 million Google Street View images with a resolution of 640 × 640 pixels collected by Yue et al. [8]. Yue et al. [8] obtained the images by sampling latitude and longitude coordinates along every 100 meters of road and used used Google Street View’s static application programming interface (API) to download images at four angles (0, 90, 180, and 270 degrees) from each sampled location.

Yue et al. [8] also created a labeled training dataset by manually annotating 18,700 images from Chicago, Illinois; Salt Lake City, Utah; Charleston, West Virginia; and a national sample from December 2016 to February 2017. This labeled dataset is used to train the classification models in this paper. Table 2 shows the details of the number of labeled images for each indicators.

### B. FLICKR DATA COLLECTION

In order to gather a dataset for our training paradigm, we obtained images related to each environment indicator from the image-sharing platform, Flickr. To collect these images, we used Flickr’s API in Python, which allows for the collection of images based on specific keywords. By providing a query keyword and a license category, the API is able to download images that fall under that specific license category.

The keywords used in our query were carefully chosen to ensure that they represent images that fall into two distinct categories: positives and negatives. The positive group includes images that contain a specific environment indicator, whereas the negative group includes images that do not contain the specified indicator. All images were obtained under the Creative Common license. Table 1 illustrates the number of images collected for each keyword.

### C. HEALTH OUTCOME DATA COLLECTION

The health data were obtained from the PLACES 2021 Release^2^ at the census-tract level. The health data include information on chronic diseases, health outcomes, health status, and preventive services across the US, derived from self-reported data in 2018 and 2019 BRFSS. The data cover various health conditions, such as obesity, high blood pressure, high cholesterol, diabetes, cancer, and depressive disorder, as well as health behaviors such as sleep and smoking. The PLACES 2021 dataset is available at the county, place, zip code, and census-tract levels [8].

## V. EXPERIMENTAL RESULTS

### A. CLASSIFICATION MODEL TRAINING

For the single-task model, we used a pretrained ResNet-18 [38] network on the ImageNet dataset [11]. The last fully connected layer was replaced with a new layer that has two outputs for binary classification. For the multi-task network, we employed the hard parameter sharing paradigm. We also used a pretrained ResNet-18 network on the ImageNet dataset. The last fully connected layer was replaced with two fully connected layers that independently predict labels for GSV images and Flickr images.

We have randomly divided both the Flickr and GSV datasets presented in Tables 1 and 2 into training and test sets with a ratio of 80:20. All images were resized to 512 × 512 and augmented using horizontal flipping and color jittering. For the single-task model, the model was trained on the labeled set of GSV images. For the multi-task model, the model was trained using both Flickr and GSV images simultaneously.

Both networks were trained using the binary cross-entropy loss function for 100 epochs with a learning rate of 1*e*^−4^. Adam [39] is used as the optimizer and we train the models with a batch size of 16.

The performance of the multi-task classifier and single-task classifier is compared based on accuracy, *F*_1_ score and balanced accuracy. The *F*_1_ score is the harmonic mean of precision and recall, with a best value of 1 and worst value of 0.


(1) $$F_{1}=2*\frac{\;\text{precision}\;*\;\text{recall}\;}{\;\text{precision}\;+\;\text{recall}\;}.$$

Balanced accuracy is used when dealing with imbalanced data and it is the arithmetic mean of sensitivity and specificity, where

(2) $$\;\text{sensitivity}\;=\frac{\text{TP}}{\text{TP}+\text{FN}},$$

and

(3) $$\;\text{specificity}\;=\frac{\text{TN}}{\text{TN}+\text{FP}}.$$

Table 2 illustrates the number of available labeled positive and negative examples for each indicator used for training. As is shown in the table, the positive and negative classes are highly unbalanced. This means that focusing solely on classification accuracy is not informative; therefore, the *F*_1_ score and balanced accuracy must be taken into consideration.

As shown in Table 3, training a multi-task classifier across different indicators results in an improvement in both the *F*_1_ score and balanced accuracy. Due to the highly imbalanced classes of the chain-link fence and streetlight indicators, there is a significant difference between accuracy and the *F*_1_ score. Multi-task learning has particularly improved the *F*_1_ scores and balanced accuracy for these indicators.

### B. REGRESSION MODELS TRAINING

**FIGURE 4.**
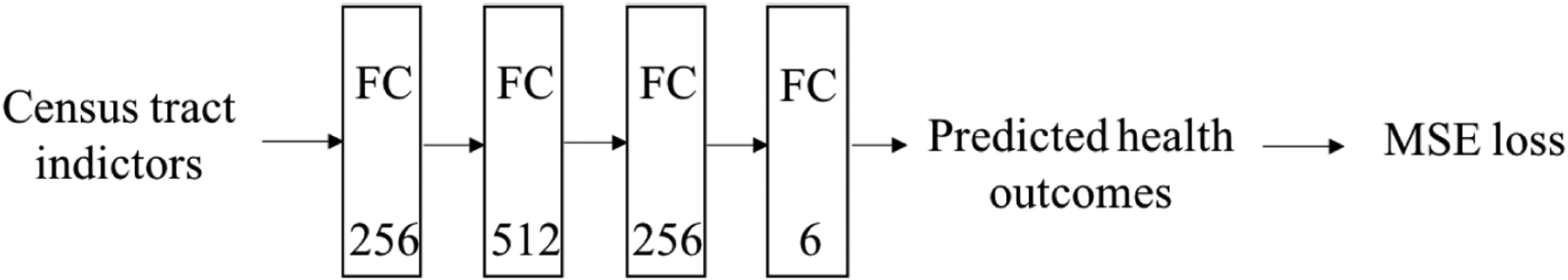
The regression neural network for estimating health outcomes.

For predicting health outcomes correlated with environment indicators, we utilized fully connected neural networks that are composed of four fully connected layers with rectified linear unit (Relu) nonlinearity and a linear output layer. Fig. 4 shows the regression neural network in detail.

At the census-tract level, 30 different health outcomes are represented and used for training the models. These outcomes include smoking, cholesterol levels, arthritis, depression, diabetes, and obesity. These health outcomes have been normalized prior to use in the model.

As explained in section III-C, the input for the regression models is a set of environment indicators represented at the census-tract level. These indicators are used in conjunction with the health outcomes, which are also represented at the same census-tract level. The number of images with a particular indicator *i* is divided by the total number of images present in the census tract *j*, *X*_*ij*_.

The regression model *f* is then trained to minimize the mean squared error (MSE) loss function:

(4) $$\text{MS}E_{i}=\frac{1}{n}\overset{n}{\underset{j=1}{\sum }}{\left|Y_{j}-f\left(X_{ij}\right)\right|}^{2},$$

where *n* is the number of census tracts, *Y*_*j*_ is the true value of health outcomes, and *f* (*X*_*ij*_) is the predicted value of health outcomes in the *j* – *th* census tract for indicator *i*.

For each predicted indicator, the performance of the model is evaluated based on *R*^2^ coefficient value:

(5) $$R_{i}^{2}=1-\frac{\Sigma_{j=1}^{n}{\left|Y_{j}-f\left(X_{ij}\right)\right|}^{2}}{\Sigma_{j=1}^{n}{\left|Y_{j}-\bar{Y}\right|}^{2}},$$

where $\bar{Y}$ is the mean of all *Y*_*j*_ values.

From the 30 different health outcomes, we selected the top two outcomes that were most correlated with each environment indicator using Pearson correlation. Selecting the top correlated health outcomes increases model’s reliability and accuracy. The model is trained to capture the most relevant information and the true relationship between the environment indicators and the health outcomes. Also, the risk of overfitting is reduced since the noise that can result from including irrelevant health outcomes is reduced.

To label the GSV test set, which consists of 164 million images, we employed both single-task and multi-task classifiers. The images were then mapped to census tracts and used for training the regression models.

The regression neural networks were trained on 34344 data points and tested on 14718 data points (70% training data, 30% testing data). The regression models were trained using the MSE loss function for 100 epochs with a learning rate of 5*e*^−4^. Adam is used as the optimizer and we train the models with a batch size of 16.

We have trained two neural network regression models and predicted health outcomes using only environment indicators predicted using single-task and multi-task learning approaches. The results are presented in Tables 4 and 5. These results confirm that the multi-task learning approach results in higher *R*^2^ values, therefore, a more accurate prediction of health outcomes compared to the single-task learning approach.

In a separate set of experiments, the regression models were controlled for demographic variables such as the percent of the population aged 65 and older, percent male, percent Hispanic, percent black, percent owner-occupied housing, and percent employed. We have trained a neural network using only the above variables to predict health outcomes and compared the results where environment indicators are also used as predictors. The predicted *R*^2^ values using the controlled variables are presented in Table 6.

**FIGURE 5.**
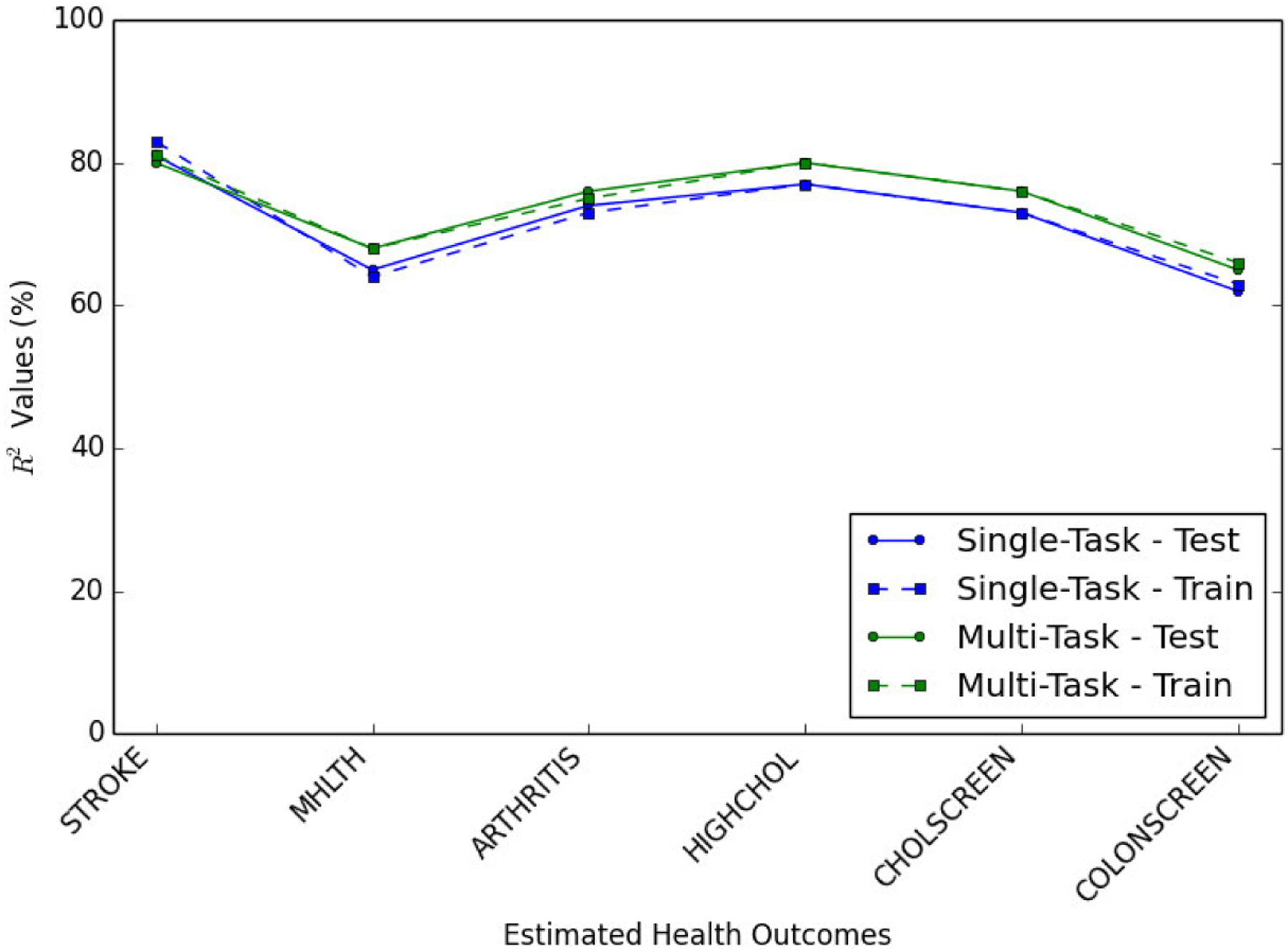
*R*^2^ values of predicted health outcomes using multi-task and single-task learning. Including the environment indicators predicted through multi-task learning improves *R*^2^ values for various health outcomes compared to single-task learning.

We have also trained separate neural networks that account for environment indicators in addition to the controlled variables. By including environment indicators, we can assess their impact on health outcomes while controlling for other variables. The results of these experiments are shown in Fig. 5, Tables 7 and 8.

Upon comparing the data presented in Tables 6 and 7, we observe that incorporating the environment indicators predicted using the single-task model does not significantly improve *R*^2^ values. Nevertheless, analysis of the results in Table 8 shows *R*^2^ values improve across different health outcomes (except for stroke rate) by including the environment indicators predicted through multi-task learning, suggesting that multi-task learning method leads to more precise predictions of environment indicators, consequently resulting in more accurate predictions of health outcomes when compared to the single-task learning approach.

After conducting the abovementioned experiments, we noticed a considerable increase in the *R*^2^ values when using demographic variables for predicting health outcomes, compared to the *R*^2^ values obtained only from environment indicators.

This difference is expected as we use indicators such as dilapidated buildings to predict health outcomes like arthritis. There are numerous variables that predict health outcomes, and we are not expecting the improvement in *R*^2^ to be significant when using environment indicators. The sociodemographic variables often predict health outcomes strongly. Social and economic status, for instance, are regarded as fundamental causes of health due to them being indicators of access to power and health-promoting resources [40].

Even though the impact of environment indicators on predicting health outcomes may be relatively small compared to sociodemographic variables, improving the detection of these indicators can still lead to an increase in the accuracy of predicting health outcomes. Environment indicators still play a role and should be addressed in predicting health outcomes. A more accurate model for identifying environment indicators leads to a more accurate estimate of health outcomes. As a result, we have a better understanding of the relationship between environment factors and health outcomes. This understanding can help us identify areas at higher risk for specific health issues and allows us to take more effective actions and targeted interventions to improve public health.

## VI. CONCLUSION

It has been demonstrated that the built environment influences health-related behaviors and outcomes. The development of an automated, cost-effective method to assess the built environment and its correlation with health outcomes would be beneficial. This automation was achieved through the use of deep learning models on Google street view (GSV) images. Our research employed a multi-task learning framework to train the deep learning model more effectively on limited labeled GSV images. Our findings confirmed that a more precise model for predicting environment indicators leads to a more accurate prediction of health outcomes.

## Figures and Tables

**TABLE 1.** Number of downloaded images from Flickr.

Indicator	Queried keyword	Number of Images
Dilapidated Building	Old/Dilapidated	37559
	Nice	21468
Chain-link Fence	Fence	32581
	Neighborhood/Street	29845
Streetlight	Street Lamp	17896
	Neighborhood	33340

**TABLE 2.** Number of labeled samples for each GSV indicator.

	Dilapidated Building	Chain-link Fence	Streetlight
Positive Class	748	1227	2144
Negative Class	999	16709	15792

**TABLE 3.** Single and multi-task learning classification results.

		Dilapidated Building	Chain-link Fence	Streetlight
Single-Task	F_1_ score	0.90	0.51	0.55
	Accuracy (%)	93.2	95.8	90.3
	Balanced accuracy (%)	91.5	69.8	69.5
Multi-Task	F_1_ score	0.95	0.57	0.59
	Accuracy (%)	93.5	96.3	91.1
	Balanced accuracy (%)	95.2	75.7	76.7

**TABLE 4.** *R*^2^ values for train and test datasets using single-task results using only environment variables. Each experiment is repeated 10 times using the bootstrap sampling method. The average and standard deviation (std) of *R*^2^ values are reported.

Estimated health outcomes	R^2^test (mean ± std)	R^2^ train (mean ± std)
STROKE	0.20 ± 7e-5	0.20 ± 2e-5
MHLTH	0.19 ± 6e-5	0.19 ± 3e-5
ARTHRITIS	0.30 ± 5e-5	0.33 ± 3e-5
HIGHCHOL	0.22 ± 6e-5	0.22 ± 3e-5
CHOLSCREEN	0.15 ± 9e-5	0.15 ± 4e-5
COLONSCREEN	0.23 ± 5e-5	0.23 ± 3e-5

**TABLE 5.** *R*^2^ values for train and test datasets using multi-task results using only environment variables. Each experiment is repeated 10 times using the bootstrap sampling method. The average and standard deviation (std) of *R*^2^ values are reported.

Estimated health outcomes	R^2^test (mean ± std)	R^2^train (mean ± std)
STROKE	0.24 ± 5e-5	0.24 ± le-5
MHLTH	0.23 ± 5e-5	0.23 ± 2e-5
ARTHRITIS	0.34 ± 9e-5	0.35 ± le-5
HIGHCHOL	0.24 ± 3e-5	0.23 ± le-5
CHOLSCREEN	0.19 ± 7e-5	0.19 ± 2e-5
COLONSCREEN	0.23 ± 3e-5	0.23 ± 2e-5

**TABLE 6.** *R*^2^ values for train and test datasets using only controlled variables. Each experiment is repeated 10 times using the bootstrap sampling method. The average and standard deviation (std) of *R*^2^ values are reported.

Estimated health outcomes	R^2^ test (mean ± std)	R^2^ train (mean ± std)
STROKE	0.81 ± 4e-5	0.82 ± 2e-5
MHLTH	0.64 ± 5e-5	0.64 ± 2e-5
ARTHRITIS	0.73 ± 6e-5	0.73 ± le-5
HIGHCHOL	0.76 ± 3e-5	0.76 ± le-5
CHOLSCREEN	0.73 ± 4e-5	0.73 ± 2e-5
COLONSCREEN	0.62 ± 6e-5	0.63 ± 3e-5

**TABLE 7.** *R*^2^ values for train and test datasets using both controlled variables and environment indicators detected using single-task learning. Each experiment is repeated 10 times using the bootstrap sampling method. The average and standard deviation (std) of *R*^2^ values are reported.

Estimated health outcomes	R^2^test (mean ± std)	R^2^ train (mean ± std)
STROKE	0.81 ± 7e-5	0.83 ± 2e-5
MHLTH	0.65 ± 4e-5	0.64 ± 3e-5
ARTHRITIS	0.74 ± 3e-5	0.73 ± 2e-5
HIGHCHOL	0.77 ± 4e-5	0.77 ± 2e-5
CHOLSCREEN	0.73 ± 5e-5	0.73 ± 3e-5
COLONSCREEN	0.62 ± 5e-5	0.63 ± 2e-5

**TABLE 8.** *R*^2^ values for train and test datasets using both controlled variables and environment indicators detected using multi-task learning. Each experiment is repeated 10 times using the bootstrap sampling method. The average and standard deviation (std) of *R*^2^ values are reported.

Estimated health outcomes	R^2^test (mean ± std)	R^2^ train (mean ± std)
STROKE	0.80 ± 6e-5	0.81 ± 2e-5
MHLTH	0.68 ± 5e-5	0.68 ± le-5
ARTHRITIS	0.76 ± 3e-5	0.75 ± le-5
HIGHCHOL	0.80 ± 3e-5	0.80 ± le-5
CHOLSCREEN	0.76 ± 4e-5	0.76 ± le-5
COLONSCREEN	0.65 ± 8e-5	0.66 ± 2e-5

## Footnotes

1
https://www.flickr.com/photos/tags/Flickr/

2
https://www.cdc.gov/places/
